# Efficacy of Intravenous Albumin for Spontaneous Bacterial Peritonitis Infection Among Patients With Cirrhosis: A Meta-Analysis of Randomized Control Trials

**DOI:** 10.7759/cureus.33124

**Published:** 2022-12-30

**Authors:** Saima Batool, Muhammad Daniyal Waheed, Kiranmayi Vuthaluru, Talha Jaffar, Sameer Krishna Prasad Garlapati, Omair Bseiso, Maira D Nousherwani, Faraz Saleem

**Affiliations:** 1 Internal Medicine, Hameed Latif Hospital, Lahore, PAK; 2 Internal Medicine, Foundation University Medical College, Islamabad, PAK; 3 Radiology, Hospital of the University of Pennsylvania, Philadelphia, USA; 4 Internal Medicine, Bahria University Medical and Dental College, Pakistan Navy Station (PNS) Shifa Hospital, Karachi, PAK; 5 Internal Medicine, Andhra Medical College, Visakhapatnam, IND; 6 Internal Medicine, Hebron University, Hebron, PSE; 7 Medicine, Shalamar Institute of Health Sciences, Lahore, PAK; 8 Internal Medicine, Akhtar Saeed Medical and Dental College, Lahore, PAK

**Keywords:** mortality, meta-analysis, spontaneous bacterial peritonitis, cirrhosis, albumin

## Abstract

Albumin is an important component in the standard therapeutic approach to spontaneous bacterial peritonitis (SBP). This meta-analysis aimed to determine the impact of intravenous human albumin in patients with cirrhosis and SBP. This study was conducted according to the guidelines of the Preferred Reporting Items for Systematic Reviews and Meta-Analyses (PRISMA). Two reviewers independently searched relevant studies using electronic databases including PubMed, Embase and Cochrane Library from the date of database inception to October 2022. The outcomes assessed in the current meta-analysis include 30-day mortality, renal impairment, changes in serum creatinine levels (mg/dl) and resolution of bacterial infection. It was found that the risk of all-cause mortality and renal impairment was significantly lower in patients receiving albumin compared to the control group. However, no significant difference was reported between the two groups in relation to changes in mean creatinine levels and resolution of infection.

## Introduction and background

Liver cirrhosis can be defined as a diffuse process with nodule formation and fibrosis that leads to certain hemodynamic changes in systemic and splanchnic circulations and impairment of liver function [1]. Every year, liver cirrhosis causes more than one million deaths worldwide and this number has been rising since the last few years [2]. Individuals with liver cirrhosis in any phase of the illness are at an enhanced risk of developing sepsis and bacterial infection [3]. The pathogenesis of bacterial infections in persons with cirrhosis is influenced by a number of factors. Immune dysfunctions associated with cirrhosis can lead to the development of all infections [4]. Translocation of certain pathological bacteria into mesenteric lymph nodes and into the blood from the intestinal lumen related to certain factors including the quality and quantity of intestinal bacterial, local defects in host immunity and increased intestinal permeability also contribute to the development of infections [5]. Bacterial infection is considered a common precipitating factor for kidney failure, acute-on-chronic liver failure and acute decompensation of cirrhosis that is characterized by multi-organ failure and acute decompensation [6-7]. All these circumstances are related to an increased risk of short-term mortality [8].

The administration of intravenous human albumin has been utilized in cirrhosis patients in various settings, such as after large-volume paracentesis, in the long-term management of decompensation and in spontaneous bacterial peritonitis [9,10]. In cases of hepatorenal syndrome, as well as in those with severe sepsis and septic shock, intravenous human albumin is given either on its own or in combination with vasoactive medications [11]. In individuals with bacterial infections and liver cirrhosis, human albumin administration can play an important role in preventing the harmful impact on renal function and circulatory function and enhancing survival [12]. Albumin's capacity to enhance intravascular volume, reduce endothelial dysfunction, and other biological features are probably connected to its positive benefits [13].

Human albumin can enhance the intravascular volume as it is a potent plasma expander [13]. Human albumin has many other characteristics, some of which are particularly significant in relation to the chronic inflammatory condition of decompensated cirrhosis. These additional characteristics are unrelated to the regulation of fluid compartmentalization [13]. These characteristics include scavenging activities and antioxidant activities because albumin is one of the main sources of extracellular sulfhydryl groups, and helps in transport and binding of several exogenous and endogenous substances and regulation of endothelial function, thus contributing to the modulation of immunological and inflammatory responses [13-14]. Certain randomized control trials (RCTs) have been conducted that investigated the effect of albumin on cirrhosis and bacterial infections, but the existing studies were underpowered for detecting the possible benefits of intravenous human albumin [15-19]. Therefore, the current meta-analysis was conducted to determine the impact of IV human albumin in patients with liver cirrhosis and spontaneous bacterial peritonitis (SBP).

## Review

Methodology

The current study was reported and carried out according to the guidelines of the Preferred Reporting Items for Systematic Reviews and Meta-Analyses (PRISMA) statement.

Search Strategy

Two reviewers independently searched relevant studies using electronic databases including PubMed, Embase and Cochrane Library from the date of database inception to October 2022. The search terms used were “albumin”, “cirrhosis”, “spontaneous bacterial peritonitis” and “efficacy”. The search included only published RCTs and was limited to human studies for all genders and geography. Trials assessing the impact of albumin in patients with cirrhosis and SBP were considered for inclusion. Ascitic fluid polymorphonuclear cell counts of 250/mm^3^ in three trials or 250/mm^3^ in one trial were used to diagnose SBP in the absence of any indication of secondary peritonitis. Two authors reviewed titles and abstracts of all the studies identified in the primary search to assess whether they were eligible for a full-text review. Full text of all eligible articles was retrieved and reviewed to assess for eligibility. Studies that assessed impacts of albumin in cirrhotic patients with non-spontaneous SBP were excluded from the current meta-analysis. In addition, we excluded reviews, retrospective studies, case report and case studies. Any disagreement between two authors was resolved via discussion or involvement of a third author. Reference lists of all selected articles were also manually search to identify additional relevant studies.

Data Extraction

Data extraction from all selected articles was done using a pre-set data extraction form developed on Microsoft Excel (Microsoft, Redmond, WA). Data extracted from selected articles included author name, year of publication, study setting, study groups, sample size, and participants’ baseline characteristics. One author extracted the data and second author cross-checked it and entered the data in Review Manager (RevMan) version 5.4.0 (Nordic Cochrane Centre, Cochrane Collaboration, Copenhagen) for data analysis.

Quality Assessment

Scoring and evaluation of included studies were based on the Jadad scoring system. The Jadad score is also known as Jadad scoring or the Oxford quality scoring system. It is a procedure utilized to independently assess the methodological quality of clinical trials. The Jadad scoring system scores range from 0 to 5, where a low score shows a poor quality study. The components of the Jadad scoring system included randomization, blinding and account of all patients. Two reviewers assessed the risk of bias independently, and differences between two reviewers were resolved via discussion.

Outcomes

The outcomes assessed in the current meta-analysis included 30-day mortality, renal impairment, changes in serum creatinine levels (in mg/dl) and resolution of bacterial infection.

Statistical Analysis

RevMan version 5.4.0 was used for data analysis to estimate the pooled risk ratio (RR) and 95% confidence interval (CI) for dichotomous outcomes and pooled mean difference (MD) and their 95% CI for continuous variables. A p-value of less than 0.05 was considered to be statistically significant. Heterogeneity among study results was assessed using I-square statistics. If the value of I-square was less than 50%, pooled estimates were calculated using the fixed effect model. Otherwise, the random effect model was used. Testing of heterogeneity was done using Cochran's Q statistics. A p-value less than 0.10 in the Cochran Q test was considered significant for heterogeneity.

Results

**Figure 1:**
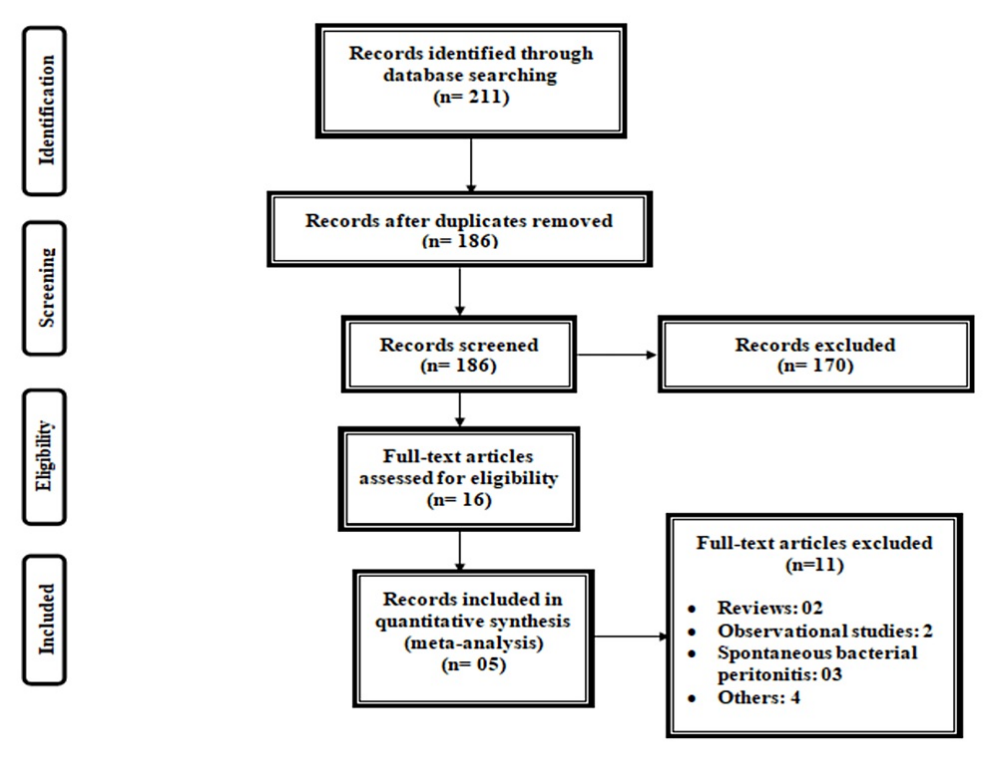
PRISMA flowchart for the selection of studies PRISMA: Preferred Reporting Items for Systematic Reviews and Meta-Analyses

Figure 1 shows the PRISMA flowchart for the selection of studies. From an initial database searching, we identified 211 studies. After removing duplicates, title and abstract screening of 186 articles was done. A total of 16 full-text studies were retrieved and reviewed for eligibility criteria. Out of these, five fulfilled the inclusion criteria and were included in the current meta-analysis. Table 1 shows the characteristics of the included studies. One study was multi-center [18], while others were conducted in a single center only [15-17,19]. Table 2 shows the quality assessment of the included studies.

**Table 1:** Characteristics of included studies NR: not reported

Author name	Year	Setting	Groups	Sample size	Mean age (years)	Males
Chen et al. [15]	2009	Single center	Albumin	15	56.5	18
			Placebo	15		
Fernandez et al. [16]	2005	Single center	Albumin	10	61	11
			Placebo	10		
Salman et al. [17]	2016	Single center	Albumin	50	52.4	84
			Placebo	50		
Sort et al. [18]	1999	Multicenter	Albumin	63	61	81
			Placebo	63		
Xue et al. [19]	2002	Single center	Albumin	56	NR	NR
			Placebo	56		

**Table 2:** Jadad scoring for quality assessment of included trials

Study	Randomized mentioned	Concealment of randomization	Blinding	Appropriate blinding method	Reporting of withdrawals	Jadad score
Chen et al. [15]	Yes	No	No	No	Yes	2
							Fernández et al. [16]	Yes	Yes	No	No	Yes	3
Salman et al. [17]	Yes	Yes	No	No	Yes	3							
							Sort et al. [18]	Yes	Yes	No	No	No	2
Xue et al. [19]	Yes	No	No	No	Yes	2							

Mortality

**Figure 2:**
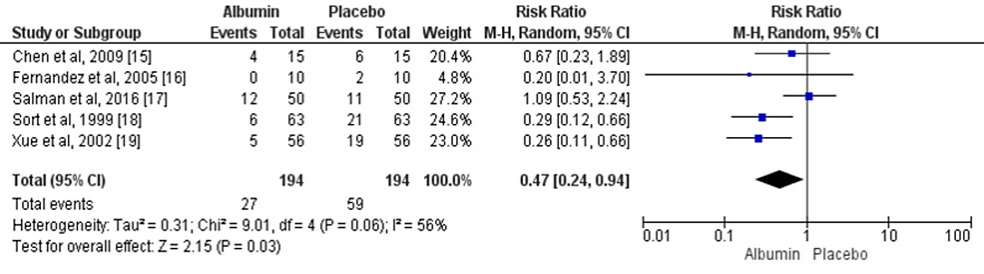
IV albumin and 30-day mortality among cirrhosis patients with spontaneous bacterial peritonitis infection M-H: Mantel-Haenszel Blue square boxes represent individual study estimates of the risk ratio and the black diamond represents pooled estimates. Source: References [15-19]

Across five trials, the pooled event rate of 30-day mortality was significantly higher in patients receiving albumin (13.91%) compared to the control group (30.41%) (RR: 0.47, 95% CI: 0.24, 0.94) as shown in Figure 2. The evidence of significant heterogeneity was found among the study results (I^2^: 56%, p-value: 0.06).

Renal Impairment

**Figure 3:**
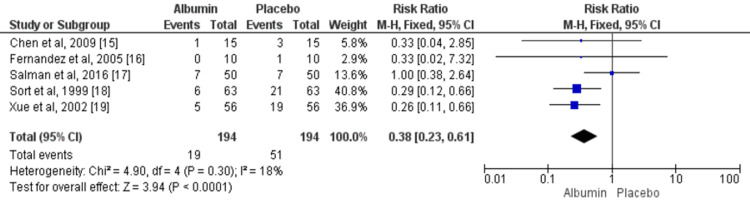
IV albumin and renal impairment among cirrhosis patients with spontaneous bacterial peritonitis infection M-H: Mantel-Haenszel Blue square boxes represent individual study estimates of the risk ratio and the black diamond represents pooled estimates. Source: References [15-19]

Five studies assessed the risk of renal impairment between patients in the albumin group and those in the control group. The pooled events of renal impairment were 9.79% and 26.28% in the albumin and control groups, respectively. The risk of renal impairment was significantly higher in patients receiving albumin compared to the control group (RR: 0.36, 95% CI: 0.23, 0.61) as shown in Figure 3. No significant heterogeneity was found among the study results (I^2^: 18%, p-value: 0.30).

Resolution of Infection

**Figure 4:**
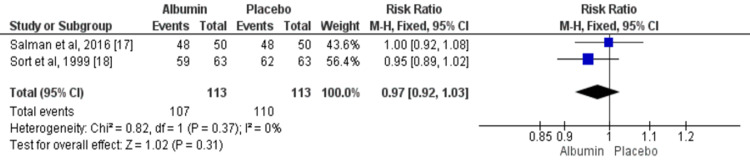
IV albumin and resolution of infection among cirrhosis patients with spontaneous bacterial peritonitis infection M-H: Mantel-Haenszel Blue square boxes represent individual study estimates of the risk ratio and the black diamond represents pooled estimates. Source: References [17-18]

Two studies compared the overall events of the resolution of infection between patients in the albumin group and control group. Overall, the resolution of infection was resolved in 94.69% patients in the albumin group and in 97.34% in the control group. No significant difference was found between two study groups in terms of resolution of infection (RR: 0.97, 95% CI: 0.92, 1.03) as shown in Figure 4. No significant heterogeneity was found among the study results (I^2^: 0%, p-value: 0.37).

Changes in Serum Creatinine Levels

**Figure 5:**
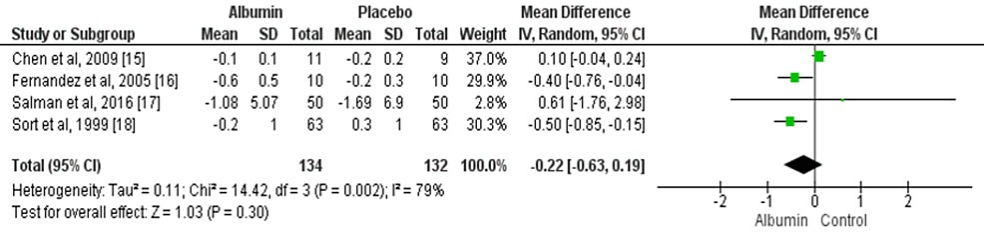
IV albumin and changes in serum creatinine levels among cirrhosis patients with spontaneous bacterial peritonitis infection Green boxes represent individual study estimates of mean difference and the black diamond represents pooled estimates. Source: References [15-18]

Four studies assessed changes in serum creatinine levels from baseline. Reduction in serum creatinine was greater in patients receiving albumin compared to the placebo group but the difference was non-significant (MD: -0.22, 95% CI: -0.63, 0.19), as shown in Figure 5. The evidence of significant heterogeneity was found among the study results (I^2^: 79%, p-value: 0.30).

Discussion

The current meta-analysis aimed to assess the impact of albumin IV infusion on mortality and renal failure in patients with liver cirrhosis and spontaneous bacterial infection. Overall, five studies were included in the current meta-analysis, including patients with SBP. In this meta-analysis, the risk of mortality and renal impairment was significantly lower in the albumin group compared to the control group. However, no significant difference was reported between the two study groups in terms of the reduction in serum creatinine and resolution of infection.

Intravenous albumin is used in patients with cirrhosis. Among cirrhosis patients with SBP, IV albumin can increase the mean arterial pressure (MAP), and decrease the serum renin and heart rate (HR), thus showing a significant improvement in the circulatory dysfunction among cirrhosis patients [16]. Moreover, cirrhosis patients receiving short-term intravenous albumin have a decreased interleukin-6 level showing an increase in systematic inflammation [16]. The mechanism through which albumin infusion enhances circulatory functions in patients with cirrhosis and SBP is by enhancing the cardiac overload and venous return [14]. However, albumin has an increased capacity to bind numerous constituents, such as bile acids, nitric oxide and cytokines [20]. Thus, it can decrease the delivery of these substances to the vascular endothelium and myocytes. In addition, albumin puts an antioxidant influence and it is known that oxidative stress can be a significant characteristic of sepsis pathogenesis [16].

The impairment of renal function is a vital clinical event in cirrhotic patients with SBP. In our meta-analysis, the risk of renal impairment was found to be significantly higher in patients receiving albumin compared to the control group. The renal impairment pathogenesis associated with SBP is possibly hemodynamic. Patients with ascites and cirrhosis have a circulatory dysfunction characterized by reduced efficient arterial blood volume, high cardiac output, hypotension, arteriolar vasodilation and enhanced circulating levels of arginine vasopressin [21-22]. The use of albumin therapy in SBP patients has been explored due to albumin's capacity to increase intravascular volume and bind inflammatory cytokines. According to the published research, albumin and antibiotics protect against renal damage, and lower mortality in SBP [23].

Two out of five included studies showed that the risk of mortality and renal impairment was significantly higher in the control group, while three studies did not demonstrate any significant difference between the two study groups. Two of these studies had a very low sample size, and thus, the power of these studies was not high enough to determine any significant difference.

The current meta-analysis also had certain limitations. First, our meta-analysis was limited by the small number of published studies. Thus, we were not able to assess publication bias and subgroup analysis via meta-regression. Second, the power of included studies was low because of the lower sample size. The American Association for the Study of Liver Diseases (AASLD) Level A recommendation that patients with SBP be managed with albumin is supported by this meta-analysis [24]. However, the current available literature is limited on outcomes in SBP patients not getting albumin along with the responsiveness of low-risk patients to albumin infusion. Therefore, future studies with a larger sample size are required.

## Conclusions

In conclusion, the risk of 30-day mortality and renal impairment was significantly lower in patients receiving albumin compared to patients in the control group. However, no significant difference was reported in relation to changes in serum creatinine levels and resolution of infection between two study groups. The meta-analysis supports the current European Association for the Study of the Liver recommendation that all patients with SBP get albumin. In addition, future studies are required to clarify the impacts of adding vasoconstrictors to plasma expanders on hemodynamics and other clinical outcomes in individuals with cirrhosis and SBP.
